# The Accelerate Pheno™ System—A New Tool in Microbiological Diagnostics of Bloodstream Infections: A Pilot Study from Poland

**DOI:** 10.3390/pathogens11121415

**Published:** 2022-11-24

**Authors:** Patrycja Zalas-Więcek, Tomasz Bogiel, Eugenia Gospodarek-Komkowska

**Affiliations:** 1 Department of Microbiology, Ludwik Rydygier Collegium Medicum in Bydgoszcz, Nicolaus Copernicus University (NCU) in Toruń, 85-094 Bydgoszcz, Poland; 2 Clinical Microbiology Division, University Hospital No. 1 in Bydgoszcz, 85-094 Bydgoszcz, Poland

**Keywords:** Accelerate Pheno™ system, bacteremia, bloodstream infections, fluorescence in situ hybridization, rapid identification and antimicrobial susceptibility testing, sepsis

## Abstract

The aim of this study was to evaluate the usefulness of the Accelerate Pheno™ system (APS) (Accelerate Diagnostics, Denver, CO, USA) for rapid laboratory diagnosis of bloodstream infections. The study included 45 positive blood samples obtained from patients hospitalized in University Hospital No. 1 in Bydgoszcz, Poland. In 40 (88.9%) blood samples, the APS was capable of identification of at least one microorganism at the genus or species level and in 38 (84.4%) of them additionally assessed antimicrobial susceptibility. The time of identification and the time to result of antimicrobial susceptibility ranged from 1:32 to 1:42 and 5:02 to 5:36 h, respectively. Six positive blood samples revealed a poly-microbial culture. In these cases, only one out of two or three microorganisms was detected by the APS, and the system assessed antimicrobial susceptibility only for them. For 78.6% positive blood samples, agreement on identification compared to mass spectrometry was found. For all but one sample, a 96–100% compliance of the resistance category was achieved when comparing the antimicrobial susceptibility testing results to conventional methods. Using the APS, the total time to report was reduced from 13:34 to even 63:47 h compared to the standard microbiological laboratory workflow. The APS is a very useful system, especially for the rapid assessment of antimicrobial susceptibility of bacteria directly from positive blood samples, offering the greatest potential for microbiology laboratories operating around the clock.

## 1. Introduction

Bloodstream infections (BSIs) account for about 10% of all healthcare-associated infections in the EU/EEA [1]. As a consequence of these infections, patients can develop sepsis, a life-threatening organ dysfunction, resulting from a host’s abnormal immune response to infection [2]. According to the European Antibiotic Resistance Monitoring Network (EARS-Net) data derived from 30 European countries and concerning 8 bacterial species isolated from blood and cerebrospinal fluid samples [3], more than half of *Escherichia coli* isolates and more than one-third of *Klebsiella pneumoniae* isolates show resistance to at least one group of antimicrobials. Moreover, multi-drug resistance (MDR) phenotypes of these isolates are often observed. The high percentage of carbapenem-resistant isolates among *K. pneumoniae* representatives (exceeding 10% in one-fourth of the countries), and an even higher percentage of carbapenem-resistant isolates among *Pseudomonas aeruginosa* and *Acinetobacter baumannii*, have been noted. There is also high percentage of methicillin-resistant *Staphylococcus aureus* (MRSA) (16.7%) and vancomycin/glycopeptides-resistant (VR/GR) *Enterococcus faecium* strains (16.8%).

Poland, compared to other European countries, has an equally alarming percentage of antibiotic-resistant strains isolated from invasive infections. Resistance to fluoroquinolones (33.0%) and third-generation cephalosporins (17.4%) are the major problems for *E. coli* strains. Meanwhile, the percentage of *K. pneumoniae* strains resistant to third-generation cephalosporins (63.0%) is one of the highest in Europe. Data showing resistance to carbapenems amongst non-fermenting rods, especially *A. baumannii* (78.2%), are also extremely worrying. The high percentages of MRSA (13.8%) and VRE/GRE (38.5%) strains are also noteworthy.

BSIs caused by antibiotic-resistant bacteria are unfortunately associated with high mortality and increased costs for the healthcare system [4]. Recent estimates based on the data from EARS-Net show that, each year, more than 670,000 infections occur in the EU/EEA due to bacteria resistant to antibiotics, and that approximately 33,000 patients die as a direct consequence of these infections [5]. The related cost to the healthcare systems of EU/EEA countries is around EUR 1.1 billion [6]. It has been considered that multidirectional activities, including antibiotic stewardship programs, enhanced hygiene, mass media campaigns, and the use of rapid diagnostic tests, can definitely improve the current situation.

Timely and accurate microbiological investigation results help clinicians to select the most appropriate antimicrobials treatment, which is crucial, especially for septic patients. Moreover, it helps to reduce the risk of microorganism transmission and the presence of outbreaks due to bacterial pathogens spread in healthcare facilities [7,8]. A performance of routine methods of microbial ID (identification) and the antibiotic susceptibility testing (AST) requires at least 24–48 h. In case of endemic areas for MDR bacteria, the complexity of the algorithm to be used increases, and the determination of the correct antimicrobial requires the addition of further rapid phenotypic tests, able to discriminate the underlying resistance mechanisms. It delays the use of targeted therapy and can result in a substantial increase of patient mortality [7,8].

One of the platforms that can quicken the diagnosis of BSIs is the Accelerate Pheno™ system (APS) (Accelerate Diagnostics, Denver, CO, USA) [9,10]. In Poland, this system has been available since August 2018. It is a new-generation, fully automated device, enabling the ID of the most common bacteria and yeasts responsible for BSIs and AST of the most common bacterial blood pathogens. ID and AST are performed directly from positive blood samples (PBSs). In this system, microbial ID relies on fully automated simultaneous fluorescence in situ hybridization (FISH)-based tests, whereas AST assessments are reported as MIC (minimal inhibitory concentration) values and are determined by measurement of morpho-kinetic changes in the cell and their growth ability, in the presence of select concentrations of antibiotics. MIC evaluation results are interpreted based on the current version of European Committee on Antimicrobial Susceptibility Testing (EUCAST) or Clinical and Laboratory Standards Institute (CLSI) guidelines [10].

Hence, the aim of this study was to evaluate the usefulness of the APS for rapid laboratory diagnosis of BSIs in a microbiology laboratory of a large centralized university hospital in Polish real-life clinical conditions.

## 2. Materials and Methods

The study included 45 PBSs (one sample per patient) obtained from patients hospitalized in Antoni Jurasz University Hospital No. 1 in Bydgoszcz, Poland, from September 2019 to March 2021. The blood samples were collected in BD BACTEC™ Plus Aerobic medium, BD BACTEC™ Lytic Anaerobic medium, or BD BACTEC™ Peds Plus medium bottles (Becton Dickinson, Franklin Lakes, NJ, USA), and firstly incubated in the BD BACTEC™ FX continuous blood culture monitoring system (Becton Dickinson) until growth was detected. The time of growth detection was recorded (Supplementary Table S3). PBSs were tested up to date (within 8 h after growth detection) with the APS (Accelerate Diagnostics, Denver, CO, USA) (version 1.4), using the Accelerate PhenoTest™ BC kit (DMIS000125B Blood Culture Kit (1.4.1.1) IVD-CE; Catalog Number: 10102028), according to the manufacturer’s instructions (Supplementary Material Tables S1 and S2). At the same time, every PBS was processed according to the standard workflow of the microbiology laboratory: a Gram-staining, a subculture on a set of solid media, and MALDI-TOF (Bruker, Billerica, MA, USA) ID. The results of the microorganism ID in MALDI-TOF fulfilled the rigorous acceptance level (an index above 2000).

For AST, the comparator for most combinations of bacteria vs. antibiotic was BD Phoenix™ M50 (Becton Dickinson), using BD Phoenix™ PMIC-88 for *Staphylococcus* spp. and BD Phoenix™ NMIC-402 for Gram-negative aerobic rods. However, the disc diffusion method (Becton Dickinson) was the comparator for testing *Enterococcus* spp. with ampicillin, vancomycin, and linezolid, and *Staphylococcus* spp. with cefoxitin. The microdilution method (MIC COL, DIAGNOSTICS s.r.o.) was the comparator for colistin. The results obtained with standard microbiological laboratory methods were used as the reference comparator for microbial ID and AST. The AST results were interpreted according to the EUCAST (version 12.0 2022) breakpoints [11]. To control the quality of AST, both with standard and APS methods, the following reference strains were used: *E. coli* ATCC 25922, *E. coli* ATCC 35218, *E. faecalis* ATCC 29212, *P. aeruginosa* ATCC 27853, and *S. aureus* ATCC 29213. The quality control results were interpreted according to the EUCAST QC (version 12.0 2022) breakpoints [12].

The study was approved by the Bioethical Commission of Ludwik Rydygier Collegium Medicum in Bydgoszcz Nicolaus Copernicus University in Toruń; Approval Code: KB 554/2019.

The time to APS report and the time to standard microbiological laboratory workflow report were calculated and compared after the growth in the BD BACTEC™ FX was detected (Supplementary Table S3), taking into account the full time needed to perform the tests and issue the results.

To determine the ID accuracy, the sensitivity and specificity were calculated by using the FISH ID probe for the APS and the results were compared to those of the standard microbiological laboratory workflow. The following formulas were used: sensitivity = (100·TP)/(TP + FN); specificity = (100·TN)/(TN + FP), where TP is the true positive, FN is the false negative, TN is the true negative, and FP is the false positive. For microorganism identified by the standard microbiological laboratory workflow, but not by the APS, the isolate was considered a false negative. For microorganisms identified by the APS, but not by the standard microbiological laboratory workflow, the isolate was considered a false positive. The AST results obtained with the application of APS were studied in terms of their % of categorical agreement (CA), defined as a percentage of the results where the resistance category (S—susceptible; I—intermediate; R—resistant) obtained by the APS was consistent with the resistance category derived from the reference method; VME (very major error), defined as a percentage of the results for which the APS reported a category S and the results of the reference method was categorized as R; ME (major error), defined as a percentage of the results where the resistance category reported by the APS was R, while with the reference method—S; and mE (minor error), defined as a percentage of the results with the APS resistance category as I and with the reference method—S or R; or the resistance category reported by the APS was S or R, while the one derived from the reference method was I. According to CLSI [13], the following criteria for comparison of the AST results were used: ≥90% for the CA rate; ≤3% for the ME and VME rates; and ≤10% for the mE rate.

## 3. Results

In 15 (33.3%) out of 45 PBSs, the APS detected *Staphylococcus* spp. and determined their AST. In five of them, the system revealed the presence of *S. aureus*, while in the remaining samples, coagulase-negative *Staphylococcus* (CNS) were noted; however, without specification to the species level. In the course of conventional diagnostics, it was found that three of these samples revealed a mixed culture of two CNSs. The time to APS report (TTR) ranged from 6:37 to 6:40 h (Table 1).

In 10 (22.2%) of the PBSs, the APS detected *Enterococcus* spp., including 8 strains of *E. faecium* and 2 belonging to *E. faecalis*. In one sample, the APS revealed alpha-hemolytic *Streptococcus* spp., without specifying to the species level and omitting an AST evaluation. The TTR ranged from 6:35 to 6:37 h (Table 2).

In 12 (26.7%) samples, the APS detected Gram-negative rods and performed AST accordingly. The APS assigned bacteria to the genus level in eight cases, and to a species level in four of them. The TTR ranged from 6:39 to 7:16 h (Table 3).

In the course of conventional diagnostics, it was found that six (13.3%) of the PBSs were poly-microbial. In these cases, only one out of two or three microorganisms was detected by the APS, and the system assessed AST only for the recognized bacteria (Table 2 and Table 3). There was only one incident where the APS identified two organisms (*E. faecium* and CNS) in a poly-microbial culture and assessed the AST for both of them (Table 4).

Due to the limitations to identify certain microorganisms using the Accelerate PhenoTest™ BC panel (Supplementary Tables S1 and S2), in three cases the APS was incapable of detection of *Clostridium septicum*, *Moraxella* spp., and *Bacteroides fragilis*. In contrast, in two PBSs, the APS failed to detect *E*. *faecium* and *E. coli*, with the following comment: ‘No ID results reported: too few cells for analysis. Recommend culture due to possibility of one or more organisms being present’ (Table 4).

The overall sensitivity and specificity for bacterial ID (per test sample), considering only on-Accelerate PhenoTest™ BC panel microorganisms, were 78.6% and 100%, respectively. The overall sensitivity and specificity, based on individual species of microorganisms, were 79.6% and 100%, respectively. However, the sensitivity and specificity, based on each group of microorganisms, were 88.2% and 100% for Gram-positive, 75.0% and 100% for Gram-negative, and 25.0% and 100% for yeasts, respectively.

The total TTR differences between APS and the standard microbiological laboratory workflow are presented in Table 1, Table 2, Table 3 and Table 4. When using APS, the total TTR was reduced from 13:34 to 63:47 h compared to the standard microbiological laboratory procedures.

Altogether, in 38 (84.4%) of the samples, the APS performed AST for at least one microorganism. A comparative analysis of AST results obtained with the APS and conventional methods used in our laboratory was carried out (Table 5 and Table 6). For the Gram-positive and Gram-negative bacteria, the CA was 99.0% and 98.3%, respectively. There was one mE for the beta-lactam antibiotics (cefepime vs. *P. aeruginosa*) and two VMEs for non-beta-lactams (one for gentamicin vs. *P. mirabilis* and one for vancomycin vs. *E. faecium*). Due to the low number of category (I) and (R) isolates, respectively, percentages of the mE and VMEs were not calculated. For the beta-lactam antibiotics, CA was 98.8%, whereas for non-beta-lactam antibiotics, CA was 98.4%. Only in the case of gentamicin was the CA, i.e., the resistant category compliance, lower than 90%; it resulted from one difference, when the APS classified *P. mirabilis* as susceptible to gentamicin, while the BD Phoenix™ M50 system categorized the strain as resistant to this antimicrobial agent.

## 4. Discussion

Due to the increasing rate of antimicrobial resistance, the susceptibility profile of the pathogens responsible especially for BSIs is relevant data. It allows for a selection of an appropriate antibiotic and avoids side effects resulting from unnecessary or inappropriate antimicrobial therapy. The rate of an inadequate initial antimicrobial therapy (IIAT) is of high relevance for the outcome of patients. The IIAT for a septic shock occurs in about 20% of patients and is associated with a five-fold reduction in a survival rate [7].

The evaluated APS enabled identification of 14 Gram-positive and 12 Gram-negative bacteria species. The advantage of the system is an additional possibility of identifying also *C. albicans* and *C. glabrata*. However, it is not possible to determine the drug susceptibility of these yeasts within this system (Supplementary Tables S1 and S2) [10]. The system enables the assessment of susceptibility to 6 and 19 antibiotics in case of Gram-positive and Gram-negative bacteria, respectively. The maximum time to ID and AST report, declared by the manufacturer, is 90 min and 5 h, respectively [10]. Moreover, the manufacturer also consecutively improves the assay to increase its relevance to microbiological investigations with respect to its potential future applications.

In our research, for 40 (88.9%) out of the 45 PBSs included into the study, the APS correctly identified microorganisms at the genus or species level. However, taking into account the microorganisms detectable by the APS and including poly-microbial cultures, for 33 (78.6%) out of the 42 PBSs included into our study, agreement on ID compared to the MALDI Biotyper^®^ (Bruker) was found. The ID compliance was slightly higher when calculated based on individual species of microorganisms (79.6%). Other authors obtained higher results consistency in this manner. Comparing the APS with the MALDI Biotyper^®^ (Bruker) and VITEK^®^ MS (bioMérieux, Marcy-l’Étoile, France) ID results, the compliance of the ID category was in the range from 87.9% to 97.1% [8,9,14,15,16,17], and 100%, respectively [18]. Thus, the APS was previously assessed as highly specific [19,20]. However, some problems with identifying microorganisms in poly-microbial blood cultures have been previously underlined. In our study, in seven PBSs, the APS identified only one out of two or three microorganisms present in the positive blood culture. Other authors also reported a correct ID only in 3 out of 6 [14], 3 out of 5 [15], 4 out of 7 [16], and 3 out of 24 [21] poly-microbial blood cultures. Taking into account the elevated cost of the test, it is worth it to use the APS for ID directly from the PBSs, but only after their initial analysis of the Gram-stain under a microscope. This approach can exclude the PBSs with the presence of poly-microbial cultures. Another approach would be to perform the direct ID of PBSs by MALDI-TOF MS method prior to APS application, if possible.

According to the CLSI criteria, a commercial AST performance should show at least 90% CA [13]. In the results of the present study, for all but one sample, a 96–100% compliance of the resistance category was achieved, when comparing the AST results obtained with the APS and conventional methods used in our laboratory (depending on the microorganisms: BD Phoenix™ M50 (Becton Dickinson), disc diffusion method, and microdilution method). Only in the case of gentamicin vs. *P. mirabilis*, the CA, i.e., the resistance category compliance, was lower than 90%. Comparing the results of the AST obtained by other authors [22] using the APS, with the results obtained with the reference method, the compliance of the AST category was found at the level of 97.9% for Gram-positive and 94.3% for Gram-negative bacteria. The APS results compared to other research on AST systems, including MicroScan Walk-Away (Beckman Coulter), VITEK^®^2 Compact (bioMérieux), and BD Phoenix™ (Becton Dickinson), gave the CA in the AST results ranging from 94.1% to 97.7% [9,15,19,21,23].

The total TTR is a crucial parameter allowing for a rapid switching from an empiric to a targeted antibiotic therapy and positively impact on patients’ outcome. In our study, the TTR of the ID result of the APS ranged from 1:32 to 1:42 h; hence, the time to final report of the AST result ranged from 6:35 to 7:16 h. With using the APS, the total TTR was reduced from 13:34 to even 63:47 h compared to the standard microbiological laboratory workflow. Comparing the APS with other ID and AST systems in terms of total TTR, it was found that microbial ID and AST results in the APS were 9:00 to 41:30 h and 24:15 to 48:15 h earlier than in the other ID and AST systems. It confirms that the APS proceeds the investigation definitely faster than conventional systems for microbial ID and AST, which was previously observed also by other researches [8,9,15,19,20,21,24,25]. Of note, the subject of five mentioned studies was to evaluate the time after which targeted antibiotic therapy was administered to patients [8,24,25,26,27]. With the use of the APS, it was possible to reduce this time by up to 30 h [27], which is of a greatest significance for the patient survival.

The APS is the first platform that delivers both microbial ID and phenotypic AST results with MIC values within 7 h, accelerating appropriate, adequate, and optimal targeted antimicrobial therapy by 1–2 days. This is especially important for septic or critically ill patients with systemic infections. Results from assessing the APS application bring the most benefits to laboratories operating around the clock and to those in which strict cooperation between microbiologists and clinicians is available [7,8,10]. The APS should be considered as a useful device for a rapid BSI laboratory diagnosis. The advantages of this system are as follows: universal panels for Gram-positive and Gram-negative bacteria and for *Candida* spp.; the simplicity to perform the testing; and the relatively large panels of antimicrobials, especially for Gram-negative bacteria. They include also new combinations of antibiotics, e.g., ceftazidime with avibactam, ceftolozane with tazobactam, and other antibiotics crucial for therapy against infections caused by MDR Gram-negative rods (e.g., colistin). Moreover, the advantage of this system is a very short time to perform the determination and obtaining the ID and reporting AST results as MIC values, which is not possible with the application of genetic methods. According to Roth et al. [28], the implementation of a rapid ID and AST method in a microbiology laboratory (including the APS) along with a well-established antimicrobial stewardship program, significantly decreases the length of a patient’s stay in a hospital, broad-spectrum antibiotic consumption, and costs to the healthcare system, which definitely fits into the program of a rational antibiotic consumption policy.

A limitation of this study is the objectively small number of tested samples. Our goal was to study the usefulness of the applied assay on actual clinical samples in real-time experiments and indicate the results of the pilot study that most probably will be continued. We proved that the test can be easily applied for standard use in a microbiology laboratory.

## 5. Conclusions

The APS is a very useful, especially for the rapid assessment of antimicrobial susceptibility of bacteria directly from PBSs, offering the greatest potential for microbiology laboratories operating around the clock.

## Figures and Tables

**Table 1:** The time to report of the identification (ID) and antimicrobial susceptibility testing (AST) results of the Accelerate Pheno™ system (APS) (part I—*Staphylococcus* spp., *n* = 15).

Test No.	Unit	ID in MALDI Biotyper	ID in APS	Time to APS ID (h:min)	Time to APS AST (h:min)	Time to APS Report (h:min)	The Total Time Difference between APS and Standard Workflow Report (d:h:min)
14133/KRCT	KAR	*Staphylococcus aureus*	*Staphylococcus aureus*	1:33	5:07	6:40	1:3:35
15875/KRCB	GER	*Staphylococcus aureus*	*Staphylococcus aureus*	1:33	5:07	6:40	1:9:44
25373/KRCB	OUM	*Staphylococcus aureus*	*Staphylococcus aureus*	1:34	5:05	6:39	1:3:33
36677/KRT	KAR	*Staphylococcus aureus*	*Staphylococcus aureus*	1:33	5:06	6:39	1:10:8
11620/KRT	KAR	*Staphylococcus aureus*	*Staphylococcus aureus*	1:33	5:06	6:39	0:18:17
24095/KRCT	CHIOE	*Staphylococcus epidermidis*	Coagulase-Negative *Staphylococcus*	1:33	5:04	6:37	0:22:13
24511/KRT	OIZ1	*Staphylococcus epidermidis Staphylococcus haemolyticus*	Coagulase-Negative *Staphylococcus*	1:34	5:03	6:37	1:0:18
24336/KRT	KCH	*Staphylococcus hominis*	Coagulase-Negative *Staphylococcus*	1:33	5:05	6:38	0:21:8
36558/KRCT	OIT	*Staphylococcus epidermidis*	Coagulase-Negative *Staphylococcus*	1:33	5:04	6:37	1:1:34
13938/KRB	KCH	*Staphylococcus epidermidis*	Coagulase-Negative *Staphylococcus*	1:34	5:04	6:38	0:20:12
12307/KRT	KAR	*Staphylococcus epidermidis Staphylococcus hominis*	Coagulase-Negative *Staphylococcus*	1:34	5:03	6:37	2:3:53
74534/KRT	OIT	*Staphylococcus epidermidis Staphylococcus haemolyticus*	Coagulase-Negative *Staphylococcus*	1:33	5:04	6:37	1:18:51
74734/KRT	NCH	*Staphylococcus epidermidis*	Coagulase-Negative *Staphylococcus*	1:32	5:05	6:37	0:23:37
53277/KRT	OIT	*Staphylococcus epidermidis*	Coagulase-Negative *Staphylococcus*	1:34	5:04	6:38	1:18:43
36558/KRCT	OIT	*Staphylococcus epidermidis*	Coagulase-Negative *Staphylococcus*	1:33	5:04	6:37	0:13:34
				1:32–1:34	5:03–5:07	6:37–6:40	0:13:34–2:3:53

CHIOE—Department of General Surgery; GER—Geriatrics Clinic; KAR—Department of Cardiology; KCH—Department of Cardiac Surgery; NCH—Department of Neurosurgery; OIZ1—Isolation (COVID-19) Department No. 1; OUM—Stroke Department; OIT—Anesthesiology and Intensive Care Unit; KRT—peripheral blood sample; aerobic growth conditions; KRB—peripheral blood sample; anaerobic growth conditions; KRCT—catheterized blood sample; aerobic growth conditions; KRCB—catheterized blood sample; anaerobic growth conditions.

**Table 2:** The time to report of the identification (ID) and antimicrobial susceptibility testing (AST) results of the Accelerate Pheno™ system (APS) (part II—*Enterococcus* spp., etc., *n* = 11).

Test No.	Unit	ID in MALDI Biotyper	ID in APS	Time to APS ID (h:min)	Time to APS AST (h:min)	Time to APS Report (h:min)	The Total Time Difference between APS and Standard Workflow Report (d:h:min)
14285/KRB	OIZ1	*Enterococcus faecium*	*Enterococcus faecium*	1:32	5:03	6:35	1:3:47
11768/KRCB	KAR	*Enterococcus faecium*	*Enterococcus faecium*	1:33	5:03	6:36	0:20:30
73865/KRT	CHIOE	*Enterococcus faecium* *Staphylococcus epidermidis*	*Enterococcus faecium*	1:32	5:03	6:35	2:1:4
36510/KRT	OIT	*Enterococcus faecium * *Staphylococcus epidermidis*	*Enterococcus faecium*	1:33	5:03	6:36	1:21:6
30827/KRCB	URO	*Enterococcus faecium * *Klebsiella pneumoniae * *Candida albicans*	*Enterococcus faecium*	1:33	5:03	6:36	3:14:41
16451/KRT	OIT	*Enterococcus faecium*	*Enterococcus faecium*	1:33	5:03	6:36	0:14:17
15500/KRT	KMS	*Enterococcus faecium * *Klebsiella pneumoniae * *Candida glabrata*	*Enterococcus faecium*	1:32	5:03	6:35	2:11:42
14516/KRB	NCH	*Enterococcus faecium*	*Enterococcus faecium*	1:34	5:02	6:36	0:14:30
23589/KRCT	OIZ2	*Enterococcus faecalis * *Acinetobacter baumannii*	*Enterococcus faecalis*	1:33	5:03	6:36	3:21:22
25159/KRT	NCH	*Enterococcus faecalis isolate#1 * *Enterococcus faecalis isolate#2 * *Staphylococcus haemolyticus*	*Enterococcus faecalis*	1:34	5:02	6:36	3:3:15
31559/KRCT	PHO	*Streptococcus viridans*, α-hem	*Streptococcus* spp.	1:37	x	1:37	-
				1:32–1:37	5:02–5:03	6:35–6:37	0:14:17–3:21:22

CHIOE—Department of General Surgery; KAR—Department of Cardiology; KMS—Department of Forensic Medicine; NCH—Department of Neurosurgery; OIT—Anesthesiology and Intensive Care Unit; OIZ1—Isolation (COVID-19) Department No. 1; OIZ2—Isolation (COVID-19) Department No. 2; PHO—Department of Pediatrics; Hematology and Oncology; URO—Department of Urology; KRT—peripheral blood sample; growth conditions; KRB—peripheral blood sample; anaerobic growth conditions; KRCT—catheterized blood sample; growth conditions; KRCB—catheterized blood sample; growth conditions.

**Table 3:** The time to report of the identification (ID) and antimicrobial susceptibility testing (AST) results of the Accelerate Pheno™ system (APS) (part III—Gram-negative rods, etc., *n* = 12).

Test No.	Unit	ID in MALDI Biotyper	ID in APS	Time to APS ID (h:min)	Time to APS AST (h:min)	Time to APS Report (h:min)	The Total Time Difference between APS and Standard Workflow Report (d:h:min)
35807/KRT	KAR	*Enterobacter cloacae*	*Enterobacter* spp.	1:34	5:36	7:00	1:1:35
66588/KRT	CHIOE	*Klebsiella oxytoca*	*Klebsiella* spp.	1:42	5:34	7:16	0:23:21
23555/KRB	KAR	*Klebsiella pneumoniae*	*Klebsiella* spp.	1:34	5:25	6:59	0:21:27
17822/KRT	NEF	*Klebsiella pneumoniae*	*Klebsiella* spp.	1:33	5:26	6:59	0:23:12
17244/KRT	KAR	*Klebsiella pneumoniae*	*Klebsiella* spp.	1:33	5:34	7:07	1:0:31
17213/KRT	OIT	*Klebsiella pneumoniae*	*Klebsiella* spp.	1:34	5:33	7:07	1:10:00
15007/KRCT	CHIOE	*Klebsiella pneumoniae * *Candida albicans*	*Klebsiella* spp.	1:34	5:36	7:00	0:22:14
24341/KRT	NCH	*Proteus mirabilis*	*Proteus* spp.	1:33	5:26	6:59	0:21:29
11542/KRCT	OIT	*Pseudomonas aeruginosa*	*Pseudomonas aeruginosa*	1:33	5:22	6:55	0:16:00
16380/KRT	PHOTS	*Pseudomonas aeruginosa*	*Pseudomonas aeruginosa*	1:33	5:23	6:56	0:22:30
14338/KRT	OIT	*Pseudomonas aeruginosa*	*Pseudomonas aeruginosa*	1:33	5:23	6:56	0:22:30
25059/KRT	OIZ2	*Acinetobacter baumannii*	*Acinetobacter baumannii*	1:32	5:07	6:39	2:15:47
				1:32–1:42	5:07–5:36	6:39–7:16	0:16:00–2:15:47

CHIOE—Department of General Surgery; KAR—Department of Cardiology; NCH—Department of Neurosurgery; NEF—Department of Nephrology; OIT—Anesthesiology and Intensive Care Unit; OIZ2—Isolation (COVID-19) Department No. 2; PHOTS—Bone Marrow Transplantation Department; KRT—peripheral blood sample; aerobic growth conditions; KRB—peripheral blood sample; anaerobic growth conditions; KRCT—catheterized blood sample; aerobic growth conditions.

**Table 4:** The time to report of the identification (ID) and antimicrobial susceptibility testing (AST) results of the Accelerate Pheno™ system (APS) (part IV—remaining, *n* = 5).

Test No.	Unit	ID in MALDI Biotyper	ID in APS	Time to APS ID (h:min)	Time to APS AST (h:min)	Time to APS Report (h:min)	The Time Difference between APS and Standard Workflow Report (d:h:min)
24999/KRCT	OIT	*Enterococcus faecium * *Staphylococcus epidermidis*	*Enterococcus faecium * Coagulase-Negative Staphylococcus	1:33	5:04	6:37	0:17:11
12658/KRCT	NEF	*Candida glabrata*	*Candida glabrata*	1:38	*-*	-	-
12878/KRB	PHO	*Clostridium septicum*	-	-	-	-	-
13545/KRCT	PHO	*Moraxella* *osloensis*	-	-	-	-	-
74436/KRCT	PHO	*Enterococcus faecium* GRE	-	-	-	-	-
66751/KRT	URO	*Escherichia coli*	-	-	-	-	-
73982/KRB	URO	*Bacteroides fragilis*	-	-	-	-	-

‘-’—lack of results in the APS; NEF—Department of Nephrology; OIT—Anesthesiology and Intensive Care Unit; PHO—Department of Pediatrics; Hematology and Oncology; URO—Department of Urology; KRT—peripheral blood sample; aerobic growth conditions; KRB—peripheral blood sample; anaerobic growth conditions; KRCT—catheterized blood sample; aerobic growth conditions; GRE—glycopeptide-resistant enterococci.

**Table 5:** Categorical agreement on bacterial susceptibility to beta-lactams determined by the Accelerate Pheno™ and BD Phoenix™ M50 systems, disc diffusion method, or microdilution method.

Antimicrobial Agent	CA	VME	ME	mE
Ampicillin	10/10	0	0	0
	100%	0.0%	0.0%	0.0%
Amoxicillin/clavulanic acid	6/6	0	0	(-) **
	100%	0.0%	0.0%	
Piperacillin/tazobactam	10/10	0	0	0
	100%	0.0%	0.0%	0.0%
Cefoxitin	14/14	-	-	-
	100%	-	-	-
Ceftazidime	10/10	0	0	0
	100%	0.0%	0.0%	0.0%
Ceftriaxone	5/5	0	0	0
	100%	0.0%	0.0%	0.0%
Cefepime	9/10	0	0	1/2
	90.0%	0.0%	0.0%	(-) ***
Ceftaroline	5/5 *	0	0	0
	100%	0.0%	0.0%	0.0%
Meropenem	11/11	0	0	0
	100%	0.0%	0.0%	0.0%
Ertapenem	7/7	0	0	(-) **
	100%	0.0%	0.0%	
Overall	87/88 (98.8%)	0	0	1

* Results for *Staphylococcus aureus* only; (-) ** European Committee on Antimicrobial Susceptibility Testing does not provide (I) category for the tested genus/species of bacteria; (-) *** due to the low number of category (I) isolates, percentage was not calculated; CA—% categorical agreement; VME—% very major error; ME—% major error; mE—% minor error.

**Table 6:** Categorical agreement on bacterial susceptibility to non-beta-lactams determined by the Accelerate Pheno™ and BD Phoenix™ M50 systems, disc diffusion method, or microdilution method.

Antimicrobial Agent	CA	VME	ME	mE
Gentamicin	6/7	1/1	0	0
	86.0%	(-) ***	0.0%	0.0%
Amikacin	11/11	0	0	(-) **
	100%	0.0%	0.0%	
Tobramycin	10/10	0	0	0
	100%	0.0%	0.0%	0.0%
Ciprofloxacin	11/11	0	0	(-) **
	100%	0.0%	0.0%	
Trimethoprim/sulfamethoxazole	13/13	0	0	0
	100%	0.0%	0.0%	0.0%
Vancomycin	24/25	1/5	0	(-) **
	96.0%	(-) ***	0.0%	
Daptomycin	14/14	0	0	0
	100%	0.0%	0.0%	0.0%
Linezolid	25/25	0	0	(-) **
	100%	0.0%	0.0%	
Colistin	11/11	0	0	0
	100%	0.0%	0.0%	0.0%
Overall	125/127 (98.4%)	2	0	0

(-) ** European Committee on Antimicrobial Susceptibility Testing does not provide (I) category for the tested genus/species of bacteria; (-) *** due to the low number of category (R) isolates, percentage was not calculated; CA—% categorical agreement; VME—% very major error; ME—% major error; mE—% minor error.

## Data Availability

The data presented in this study are available on request from the corresponding author.

## Supplementary Materials

The following supporting information can be downloaded at: https://www.mdpi.com/article/10.3390/pathogens11121415/s1, Table S1: Gram-positive bacteria and yeasts detectable by the Accelerate Pheno™ system, the corresponding antimicrobials included in their antimicrobial susceptibility testing and selected resistance mechanisms; Table S2: Gram-negative bacteria detectable by the Accelerate Pheno™ system, the corresponding antimicrobials included in their antimicrobial susceptibility testing and selected resistance mechanisms; Table S3: The pre-incubation times of blood samples (*n* = 45) in BD BACTEC™ FX.
