# Supplementary material for: The Accelerate Pheno™ System—A New Tool in Microbiological Diagnostics of Bloodstream Infections: A Pilot Study from Poland
Source: Pathogens. 2022 Nov 24;11(12):1415. doi: 10.3390/pathogens11121415 (PMC9781321; doi:10.3390/pathogens11121415)
Supplement: Supplementary file 1 [file pathogens-11-01415-s001.zip › pathogens-2003621-supplementary.pdf]

Table S1. Gram-positive bacteria and yeasts detectable by the Accelerate Pheno™ system, the corresponding antimicrobials included in their antimicrobial susceptibility testing and selected resistance mechanisms

**Gram-positive bacteria**	**Identification**	**Methicillin resistance**	**Ampicillin**	**Ceftaroline**	**Vancomycin**	**Linezolid**	**Daptomycin**	**Trimethoprim/ sulfamethoxazole**
Staphylococcus aureus	•	•		•	•	•	•	•
Staphylococcus lugdunensis	•	•		•	•	•	•	•
Staphylococcus capitis<br>*Staphylococcus epidermidis*<br>*Staphylococcus haemolyticus*<br>*Staphylococcus hominis*<br>*Staphylococcus warneri*	•[1]	•			•	•	•	
Streptococcus agalactiae	•							
Streptococcus gallolyticus<br>*Streptococcus mitis*<br>*Streptococcus oralis*<br>*Streptococcus pneumoniae*	•[2]							
Enterococcus faecalis	•		•		•	•	•	
Enterococcus faecium	•		•		•	•	•	
**Yeasts**								
Candida albicans	•							
Candida glabrata	•							

[1] identified as Coagulase-Negative *Staphylococcus* spp.
[2] identified as *Streptococcus* spp.
• detectable

Table S2. Gram-negative bacteria detectable by the Accelerate Pheno™ system, the corresponding antimicrobials included in their antimicrobial susceptibility testing and selected resistance mechanisms

**Gram-negative bacteria**	**Identification**	**AmpC screen**	**Ampicillin**	**Amoxicillin/ clavulanic acid**	**Piperacillin/ tazobactam**	**Ceftazidime/ avibactam**	**Ceftolozane/ tazobactam**	**Cefazolin**	**Cefuroxime**	**Ceftriaxone**	**Ceftazidime**	**Cefepime**	**Aztreonam**	**Meropenem**	**Ertapenem**	**Gentamicin**	**Tobramycin**	**Amikacin**	**Trimethoprim/ sulfamethoxazole**	**Ciprofloxacin**	**Colistin**
Escherichia coli	•	•	•	•	•	•	•	•	•	•	•	•	•	•	•	•	•	•	•	•	•
Klebsiella pneumoniae<br>*Klebsiella oxytoca*<br>*Klebsiella aerogenes*	•	•		•	•	•	•	•	•	•	•	•	•	•	•	•	•	•	•	•	•
Proteus mirabilis<br>*Proteus vulgaris*	•	•		•	•	•	•		•	•	•	•	•	•	•	•	•	•	•	•	
Enterobacter spp.	•				•	•	•			•	•	•	•	•	•	•	•	•	•	•	•
Citrobacter freundii<br>*Citrobacter koseri*	•	•		•	•	•	•		•	•	•	•	•	•	•	•	•	•	•	•	•
Serratia marcescens	•				•	•	•			•	•	•	•	•	•	•	•	•	•	•	
Pseudomonas aeruginosa	•				•	•	•				•	•	•	•		•	•	•		•	•
Acinetobacter baumannii	•				•							•		•				•	•	•	•

• detectable

Table S3. The pre-incubation times of blood samples (n=45) in BD BACTEC™ FX

Test No.	The time of BD BACTEC™ FX pre-incubation (d:h:min)	Test No.	The time of BD BACTEC™ FX pre-incubation (d:h:min)	Test No.	The time of BD BACTEC™ FX pre-incubation (d:h:min)
14133/KRCT	1:02:19	14285/KRB	0:08:45	17244/KRT	0:11:40
15875/KRCB	0:07:13	11768/KRCB	0:16:19	17213/KRT	0:07:53
25373/KRCB	0:08:38	73865/KRT	0:09:10	15007/KRCT	1:14:38
36677/KRT	0:09:48	36510/KRT	0:11:45	24341/KRT	0:10:46
11620/KRT	0:23:34	30827/KRCB	0:05:58	11542/KRCT	0:15:45
24095/KRCT	0:18:03	16451/KRT	0:22:01	16380/KRT	0:14:56
24511/KRT	0:18:09	15500/KRT	0:09:08	14338/KRT	0:13:37
24336/KRT	1:12:39	14516/KRB	0:16:51	25059/KRT	0:13:14
36558/KRCT	1:04:30	23589/KRCT	0:14:15	24999/KRCT	0:15:34
13938/KRB	0:18:21	25159/KRT	0:13:15	12658/KRCT	1:00:53
12307/KRT	0:15:03	31559/KRCT	1:17:43	12878/KRB	0:11:21
74534/KRT	0:17:33	35807/KRT	0:12:13	13545/KRCT	1:18:59
74734/KRT	0:08:25	66588/KRT	0:10:33	74436/KRCT	0:14:39
53277/KRT	1:18:00	23555/KRB	0:15:57	66751/KRT	0:02:05
36558/KRCT	1:04:30	17822/KRT	0:07:03	73982/KRB	0:20:35
